# Planetary-Scale Geospatial Open Platform Based on the Unity3D Environment

**DOI:** 10.3390/s20205967

**Published:** 2020-10-21

**Authors:** Ahyun Lee, Yoon-Seop Chang, Insung Jang

**Affiliations:** City and Geospatial ICT Research Section, Electronics and Telecommunications Research Institute (ETRI), 218 Gajeong-ro, Yuseong-gu, Daejeon 34129, Korea; ychang76@etri.re.kr (Y.-S.C.); e4dol2@etri.re.kr (I.J.)

**Keywords:** geographic information systems, terrain mapping, 3-D map

## Abstract

Digital twin technology based on building a virtual digital city similar to a real one enables the simulation of urban phenomena or the design of a city. A geospatial platform is an essential supporting component of digital twin cities. In this study, we propose a planetary-scale geospatial open platform that can be used easily in the most widely used game engine environment. The proposed platform can visualize large-capacity geospatial data in real time because it organizes and manages various types of data based on quadtree tiles. The proposed rendering tile decision method provides constant geospatial data visualization according to the camera controls of the user. The platform implemented is based on Unity3D, and therefore, one can use it easily by importing the proposed asset library. The proposed geospatial platform is available on the Asset Store. We believe that the proposed platform can meet the needs of various three-dimensional (3-D) geospatial applications.

## 1. Introduction

A geospatial platform is the first step toward understanding the real world as a digital world. A digital twin is a digital model of a physical entity [1,2]. It virtually simulates and predicts simulation results by creating digital twins of objects [3]. Digital twins are attracting attention as a technology that can be used to address various industrial and social problems and that has applications in manufacturing. A three-dimensional (3-D) geospatial platform is an essential part of a digital twin city. It can digitally organize and visualize terrains, buildings, and other structures [4,5]. Virtual Singapore [6] is a representative example of a simulation of a real city using digital geospatial data. This simulated city can be used to study various urban phenomena and urban design scenarios.

**Figure 1:**
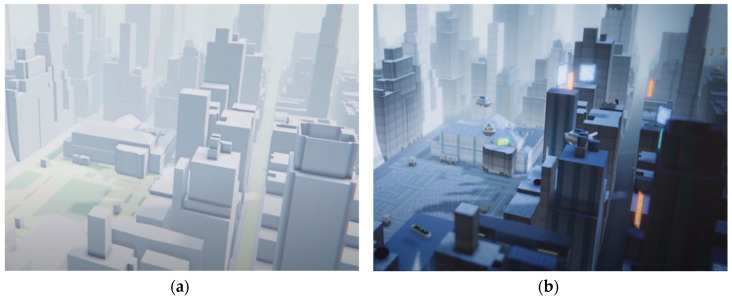
Examples of city models developed using game solution of Google Maps platform [15]: (**a**) initial geospatial model from Google Maps platform; (**b**) model modified and improved augmented by a user.

Web-based geospatial platforms offer excellent user accessibility and convenience. We can use Google Earth to explore anywhere in the world with a web browser [7,8]. Cesium allows users to upload their own 3-D geospatial data to create and expand 3-D city models [9]. These web-based platforms only require a web browser that supports web standards and WebGL; they do not require the installation of additional libraries or programs [10,11]. However, they have functional limitations as web browsers. They are limited in their ability to provide functionality that the platform does not support. In addition, the execution environment has performance constraints as a web browser. On the other hand, standalone geospatial platforms make it possible to use high-quality data and functions in a high-performance computing environment, although user accessibility is limited [12,13,14]. The game solution of Google Maps platform can use Google’s geospatial data based on the real world to build a 3-D environment model to suit a user’s purposes [15]. Figure 1a shows a city model created by importing Google geospatial data into a blank project. Figure 1b shows a city model that has been modified and augmented by the user. The ArcGIS City Engine [16], a 3-D modeling software package that allows a user to design a city, is the software that is the most widely used to create large-scale responsive and immersive urban environments based on geospatial data.

The purpose of our proposed geospatial platform differs from that of the aforementioned standalone GIS platforms, wherein a user creates a new city model on the basis of real geospatial data. Our platform is similar to the web-based Google Earth—the only difference is that ours is built on the basis of a game engine instead. Moreover, our platform involves data structure and management functions that support the use of geospatial data in various ways. It displays the user-generated geospatial data in real time on a planetary-scale. There exists a game solution of Google Maps platform based on the game engine that provides geospatial data, where users use geospatial models as shown in Figure 1a. The difference, however, between Google Maps and our proposed platform lies in the intended purpose of the platform. Google Maps aims to bring the geospatial data built by Google into the game engine. The user can then edit it as shown in Figure 1b [15,17]. On the contrary, our proposed platform was developed for the purpose of tiling-based geospatial data management for rendering on a planetary-scale. Therefore, the functions of our platform, which is based on the game engine, resemble the data management and visualization functions of the web-based Google Earth.

We selected Unity3D, the most widely used game engine in the world, to build our proposed geospatial platform, because of Unity3D’s advantages of convenience, accessibility, and extensibility of external libraries. Unity3D is capable of debugging various execution environments and is suitable for use in developing applications for various purposes [18,19,20]. Unity3D currently supports more than 25 multi-platforms; more than 60% of the development of mixed reality or virtual reality devices that can maximize user concentration is progressing with the aid of Unity3D [21].

**Figure 2:**
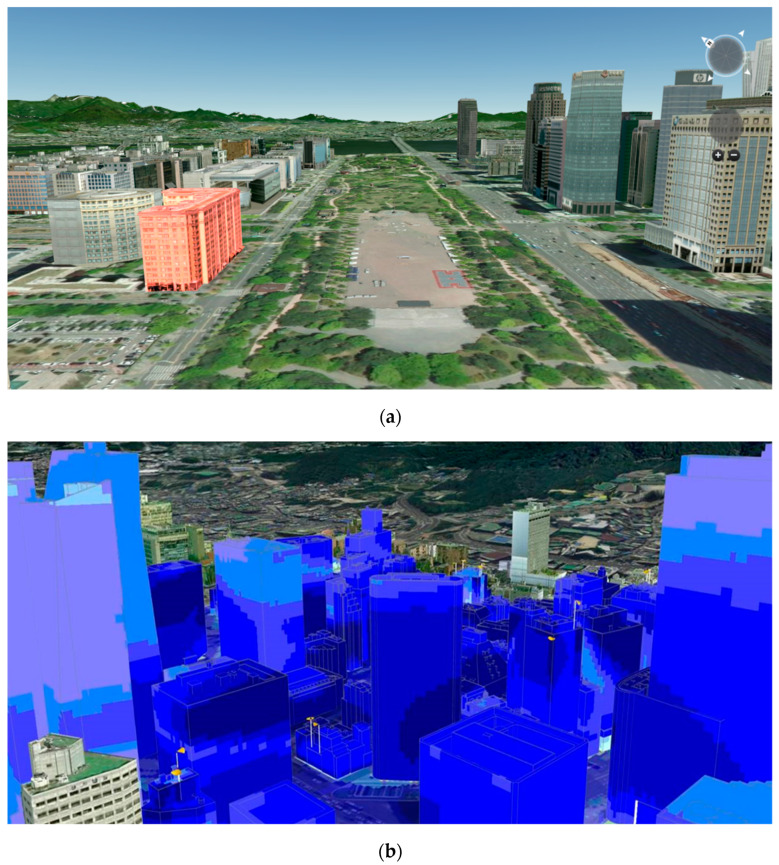
Web-based VWorld geospatial data: (**a**) VWorld service site [22]; the selected building is shown in red; (**b**) 5G radio wave environment and antenna visible area analysis using VWorld data.

Some countries and cities have been collecting static geospatial data, such as terrain, building, and road data, for use on a digital twin city platform. They either choose an existing platform or develop a new platform, depending on the purpose of each digital twin city platform. VWorld contains the most precise geospatial data in South Korea [22]. It is similar to the web-based Google Earth; however, the terrain and building models are managed separately, as shown in Figure 2a. All the building models created as individual models are obtained via DataAPI [23]. As a high-resolution building model is created separate from the terrain, it requires more large-scale data when compared to the data required by Google Earth. However, simulations based on precise city models can be obtained. SK Telecom, a Korean telecommunications company, analyzed the 3-D stereoscopic information-based radio wave environment and the 5G antenna visible area analysis for a 5G wireless network design using the VWorld data, as shown in Figure 2b. This showcases the application of a digital twin city platform in which high-precision geospatial data are built.

The VWorld map service provides two types of geospatial information platforms. Anyone can easily use a geospatial map through a WebGL platform based on web standards. The standalone platform provides various user-customizable functions and supports for creating applications [24,25,26]. However, a user can only develop the functions supported by the platform. For example, it is possible to add 2-D/3-D markers, 2-D/3-D polygons, texts, etc., at specific locations. It is not possible, however, to develop applications for a special scenario, such as a satellite observation simulation or an autonomous driving simulation [27]. Development of a digital twin city platform requires convenient support for special scenarios. The platform can support different geospatial data formats. Our goal was to develop a universal digital twin city platform that can express geospatial data via real-time streaming and meet the specific needs of a digital city platform.

**Figure 3:**
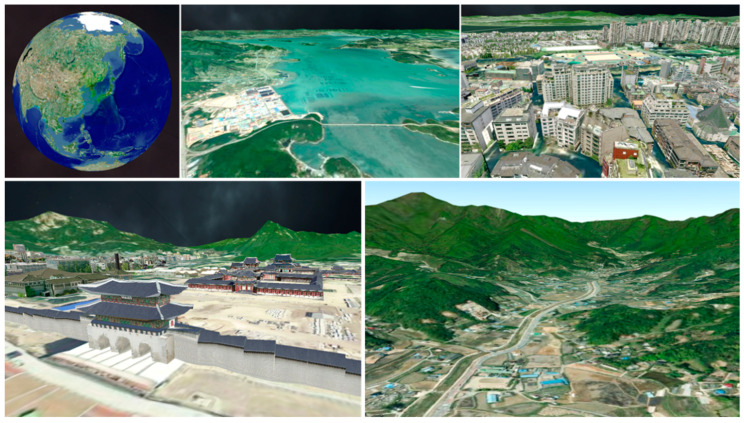
Game window examples of the proposed geospatial platform.

In this paper, we present a geospatial platform prototype based on the universal game engine Unity3D, as shown in Figure 3. The remainder of this paper is structured as follows. In Section 2, we describe the structure of the proposed platform based on Unity3D. In Section 3, we explain how to organize and manage geospatial data based on the quadtree tile structure. A validation experiment and analysis of its results are discussed in Section 4. Finally, we present concluding remarks in Section 5.

## 2. Methods: Unity3D-Based Platform

**Figure 4:**
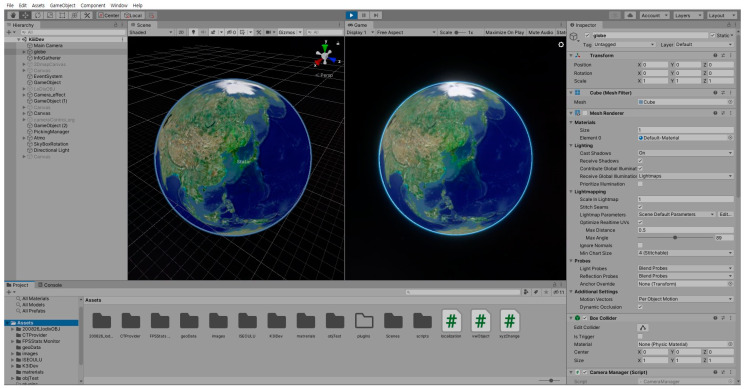
Proposed geospatial platform in the game engine editor.

The proposed geospatial platform allows users to easily build a geospatial application without geospatial expertise. The setup process is as simple as downloading the asset library and importing it into your Unity3D project, as shown in Figure 4. The platform enables the development of various geospatial applications through utilities and external assets in Unity3D.

**Figure 5:**
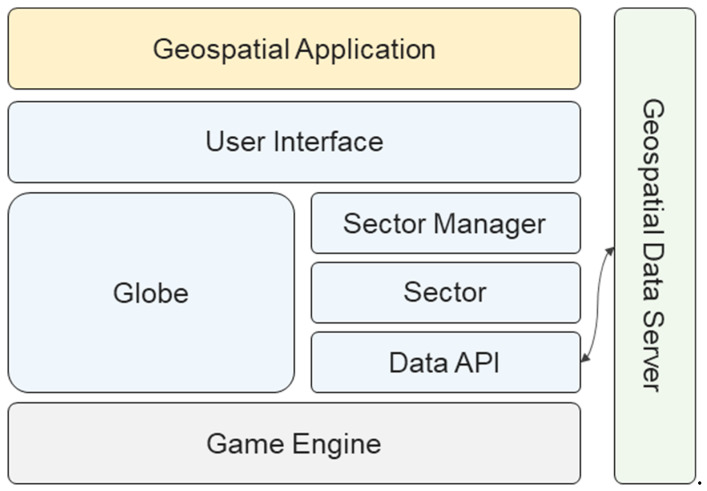
Proposed platform structures. The blue blocks pertain to the proposed asset library in the game engine.

Figure 5 shows the proposed platform structure. The proposed open platform was implemented based on Unity3D. The *Globe* class is the 3-D global model. The Earth model consists of 71.5 billion tile-based terrain models. The *Sector* class is a unit that contains a tile, and various types of geospatial data are included in the tile. Data for composing the sectors were requested from the external geospatial data server using the *Data API*. The platform uses the VWorld Data Server API [22]. The *Sector Manager* class manages the 71.5 billion tile-based sectors. The *User Interface* class includes the map movement and direction control, according to the geospatial characteristics of the globe. A user can develop a geospatial application by importing the proposed asset library indicated by the blue blocks into the empty project in Unity3D.

### 2.1. Camera Control

**Figure 6:**
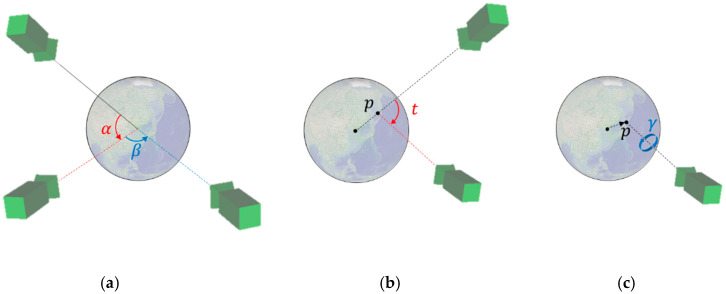
Camera control for the three-dimensional (3-D) globe-based map: (**a**) rotation based on latitude and longitude; (**b**) tilt transformation at the intersection point p between the camera’s principal line and the surface of the globe; (**c**) rotation based on the camera principal line.

The camera control provided with the *User Interface* class has movements for observing geospatial data expressed as a globe [28]. It has transformations such as rotation, movement, and tilt, depending on mouse events. First, the camera has a rotational movement according to the latitude and longitude when the principal line of the camera is fixed towards the center point of the globe. Figure 6a shows an example of camera movements according to the rotations around the latitude and longitude. The red α is the latitude change and rotates around the right vector of the camera at the center point of the globe. The blue β is the longitude change and rotates around the up vector of the camera at the center point of the globe. When the left mouse button is pressed, the vertical movement of the cursor determines the angle in latitude, and the horizontal movement of the mouse cursor determines the angle in longitude.

(1) angle=delta/s·elev/R/UR

*delta* denotes the amount of pixel-based movement of the mouse cursor on the 2-D screen. The movement sensitivity can be set according to the size of *s* in (1). *elev* is the elevation of the camera position calculated relative to the surface of the globe. *R*, the radius of the Earth, is 6,387,137 m, and *UR* is the radius value used on the proposed platform. The transform property of the game object uses the float type in Unity3D. In the case of the float type, if it exceeds the limit that can be expressed by significant-digit bits, it takes an approximate value. When a planetary-scale map is used, floating point precision limitations can arise in Unity3D. Some models with large position coordinates are not normally displayed, as they are too far from the origin of the game coordinate system. The proposed platform sets the radius as 10,000 m, which is smaller than the actual radius 6,387,137 m. Depending on the application, *UR* can be set to a user-defined value. Other property values can be defined in the inspector window of the *Globe* game object.

**Figure 7:**
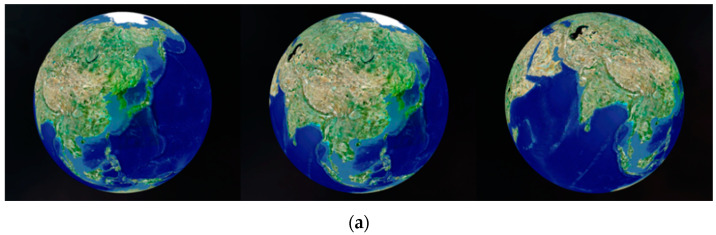

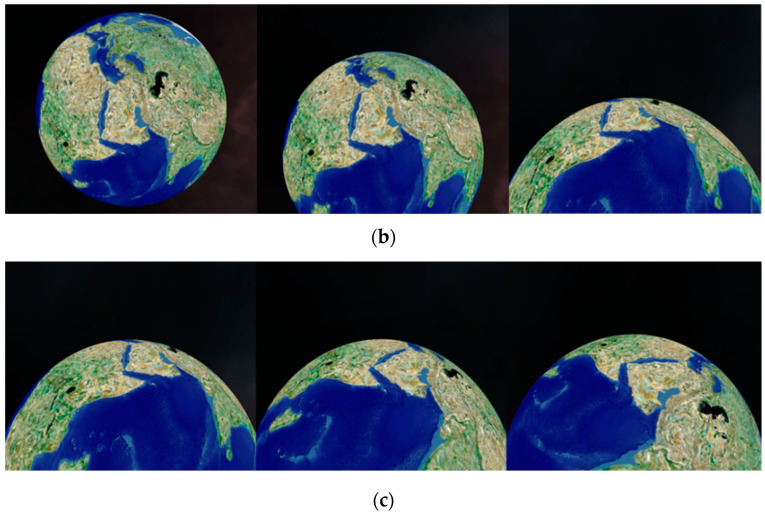
Examples of the camera control: (**a**) rotation based on latitude and longitude in Figure 6a; (**b**) tilt transformation in Figure 6b; (**c**) rotation based on the camera principal line in Figure 6c.

Figure 6b shows an example of the tilt transformation. The tilt transformation rotates on the right vector of the camera at the intersection point p between the camera’s principal line and the surface of the globe. When the tilt angle is 0°, the camera’s principal line points toward the center point of the globe. When the tilt angle changes, the camera’s principal line does not face the center point of the globe, and the direction of the camera can be adjusted by looking away from the surface of the globe, as shown in Figure 3. The vertical movement of the cursor determines the tilt angle while the right mouse button is pressed. The horizontal change of the mouse cursor controls the blue γ rotation. The rotation of γ occurs on the *p* vector at the intersection point *p*, as shown in Figure 6c. Figure 7 shows example motions of the proposed camera control. The translation and rotation of the camera are similar to the motion of a satellite.

### 2.2. Positioning Geospatial Model

**Figure 8:**
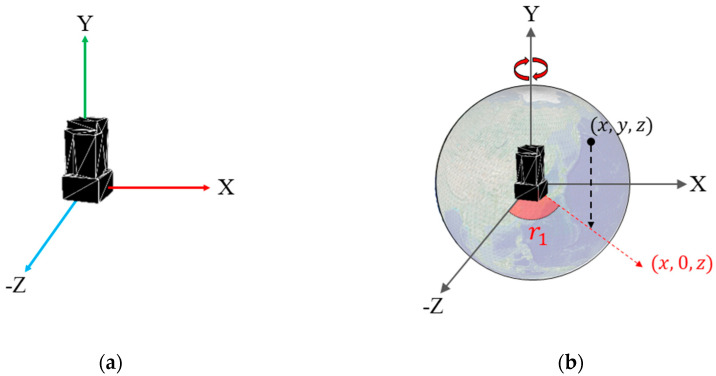

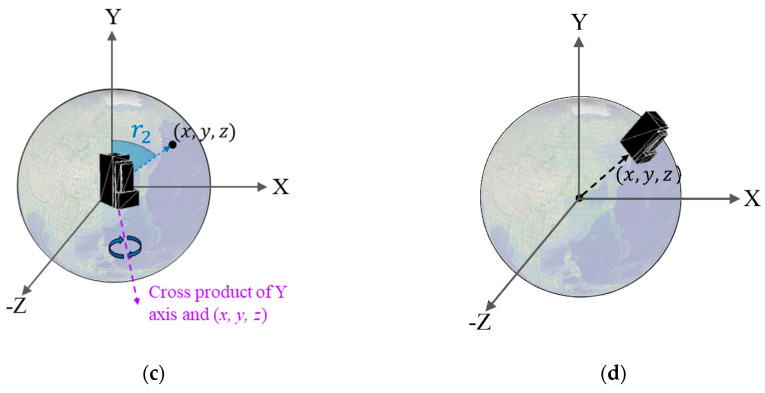
Positioning 3-D geospatial model: (**a**) initial position and orientation of the 3D model; (**b**) rotation $r_{1}$ on the Y-axis; (**c**) rotation $r_{2}$ on the cross product of (x, y, z) and the Y-axis; (**d**) result of positioning 3-D geospatial model.

On the proposed platform, we define the 3-D location of geospatial data items in terms of latitude, longitude, and elevation. The coordinate system of the proposed platform is left-handed. The Y-axis points in the head-up direction. The 3-D model source is placed perpendicular to the origin on the Y-axis, as shown in Figure 8a. The lowest value of a building is 0 on the Y-axis. The Z axis is the south side of the building, where the building will be placed on the Earth’s surface. The direction of the Z-axis points toward the South Pole when the building is positioned on the surface of the globe.

A 3-D model is assumed to be placed in advance at a position and orientation, as shown in Figure 8a, before the proposed positioning method is applied. Using the method, the 3-D model, with latitude, longitude, and elevation data, can be positioned perpendicular to the surface of the globe, as shown in Figure 8d. The latitude, longitude, and elevation of the model are converted into 3-D coordinates in the proposed 3D geospatial platform as follows:

(2) h=UR+elev·UR/R

(3) $$\left(\begin{array}{c} x\\ y\\ x \end{array}\right)=\left(\begin{array}{c} h*\cos lat\cdot \sin lon\\ h\cdot \sin lat\\ -h*\cos lat\cdot \cos lon \end{array}\right)$$

**Figure 9:**
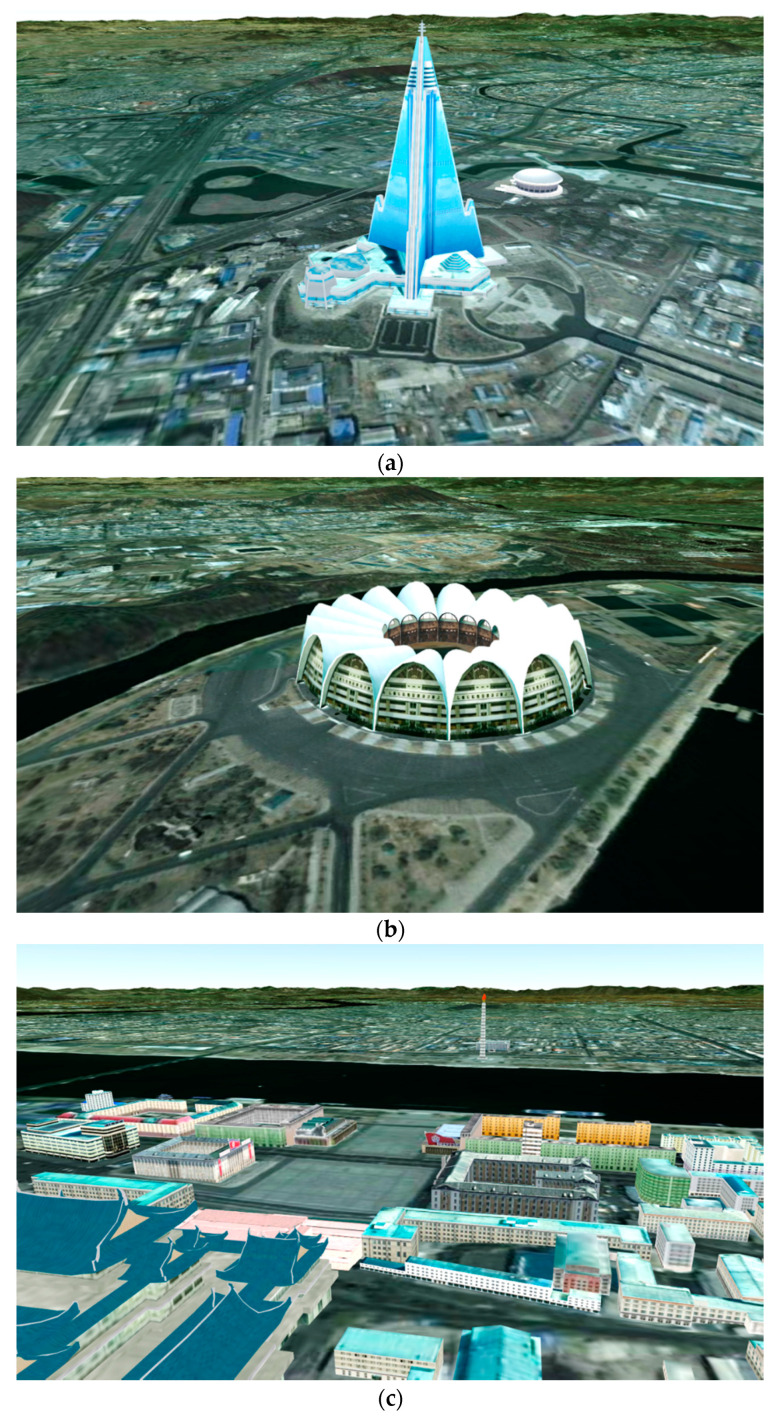
Rendering results for representative buildings in Pyongyang, North Korea on the proposed platform: (**a**) Ryukyung Hotel; (**b**) Neungrado Stadium; (**c**) Kim Il Sung Square.

The Earth is assumed to be a complete sphere, not an ellipsoid. *Lat* signifies latitude, *lon* signifies longitude, and *elev* signifies elevation. The initial 3-D source model illustrated in Figure 8a is transformed on the surface, as shown in Figure 8d, through two rotations and one translation. $r_{1}$ is the angle between the (x, 0, z) vector and the Z-axis, whose value on the Y-axis is 0, as shown in Figure 8b. The source model first rotates $r_{1}$ degrees around the Y-axis. $r_{2}$ is the angle between the (x, y, z) vector and the Y-axis, as shown in Figure 8c. The source model then rotates $r_{2}$ degrees around the cross product of (x, y, z) and the Y-axis. The model perpendicular to the Y-axis rotates in a form perpendicular to the surface of the model’s location through $r_{1}$ and $r_{2}$ rotations. Finally, the source model translates at (x, y, z), as shown in Figure 8d. As a result of these two rotations and one translation, the source model is located in position with the latitude, longitude, and elevation of the model, as shown in Figure 9.

## 3. Methods and Results: Data Management and Rendering

The geospatial data used in the proposed platform are the VWorld data that have been assembled for most cities and high-precision data in South Korea [22]. VWorld provides aerial images of the whole world, and provides aerial images within 12 cm accuracy for the Korean Peninsula (South and North Korea). VWorld has 3-D buildings and structure models for major cities in South Korea, such as Seoul, Busan, Incheon, and Daejeon. It also has 3-D buildings and structures for some representative buildings of Pyongyang in North Korea, such as the Ryukyung Hotel and the Neungrado Stadium, as shown in Figure 9. This section describes how the geospatial data that fit the structure of the VWorld tiles are managed. We also propose a method for visualizing the geospatial data provided by the VWorld server, which has a total capacity of 30 TB or more in real time.

### 3.1. Sector

**Figure 10:**
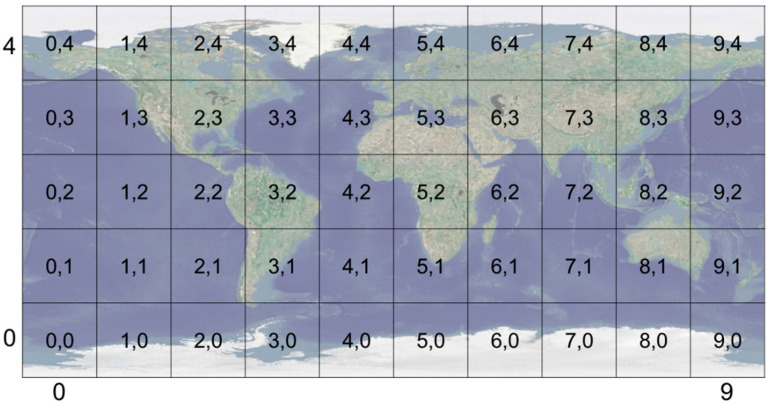
Quadtree-based tile structure (IDX, IDY): Level 0 tiles consist of 50 tiles of the Earth’s surface.

**Figure 11:**
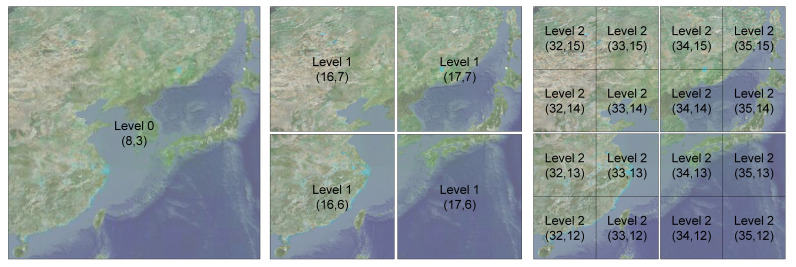
Example division of quadtree-based tiles (IDX; IDY): a level 0 tile (latitude: 18°–54°, longitude: 108°–144°, South Korea) is divided into four level 1 tiles or 16 level 2 tiles. (**a**) level 0 tile; (**b**) four level 1 tiles; (**c**) 16 level 2 tiles.

On the proposed platform, all formats of geospatial data are visualized as quadtree-based tiles [29,30,31]. VWorld tiles consist of tiles from level 0 to level 15, with 16 steps in total. The surface of the globe is divided into five latitude and ten longitude steps into 36 tiles at level 0, as shown in Figure 10. As the camera approaches the surface of the globe, a tile is divided into four tiles, and the level of the tile is increased by one. Figure 11 shows an example in which a tile at level 0 is divided into four tiles at level 1 or 16 tiles at level 2. All 3-D terrain models have an aerial image with 256 × 256 resolution and a 64 × 64 × 2 mesh model. As the level of the tiles increases, the representation of the 3-D terrain model becomes more detailed.

The level of a displayed tile is determined by the distance between the camera and the terrain model of the tile. When the distance between the camera and the terrain increases, the low-level tiles are represented [32]. However, when the distance decreases, high-level tiles are represented. Equation (4) is a method for determining the rendering level. *c* is the center position of the camera, and *s* is the center position of the terrain model of the tile.

(4) $$level=\lceil \frac{\log(R/\sqrt{{\left(c_{x}-s_{x}\right)}^{2}+{\left(c_{y}-s_{y}\right)}^{2}+{\left(c_{z}-s_{z}\right)}^{2}}}{\log2}\rceil$$

**Figure 12:**
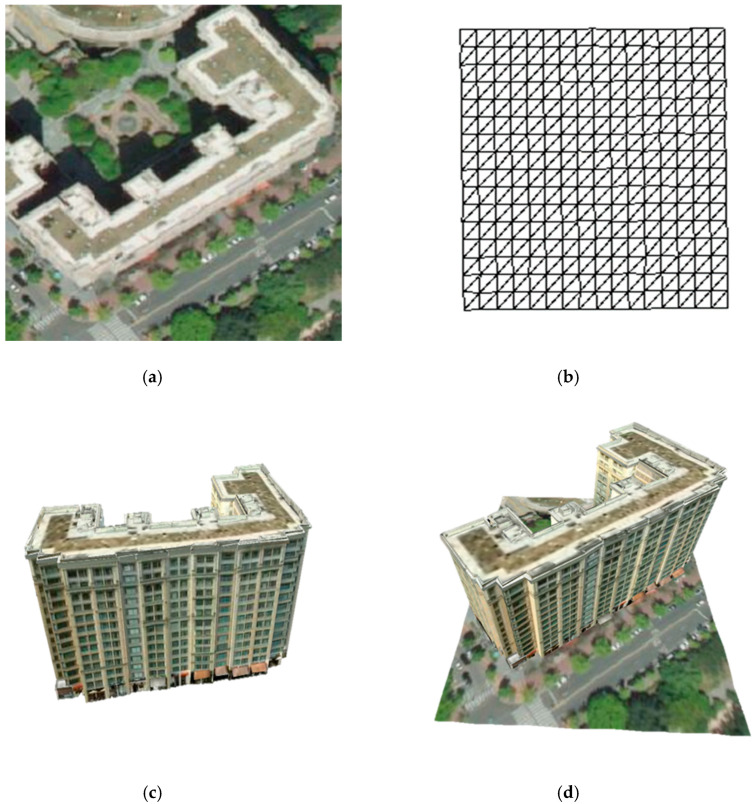
Examples of a sector composition: (**a**) aerial image; (**b**) 3-D terrain mesh model generated from digital elevation model (DEM); (**c**) 3-D building model; (**d**) rendering result of geospatial data in a sector.

A sector is a unit that contains various types of geospatial data in a tile area. Geospatial data located in the latitude and longitude areas of the tile are grouped into the same sector. Each type of geospatial data in a sector is managed as a layer. For example, the active layers are 3-D terrain models and 3-D buildings. When a sector is created, the proposed platform requests that the 3-D terrain and 3-D building model data, which are included in the activated layer, be provided to the external data server. When the request and creation of the sector are completed, it can be the target of rendering. Figure 12 shows an example of a sector composition. The terrain model is created using an aerial image as a texture source image and a 3-D terrain mesh model generated from a digital elevation model (DEM). Various geospatial data types, such as building, facility, and local name data, are included in a sector based on the terrain model. A sector is uniquely defined as (level, IDX, IDY). An index for sector identification is defined as follows:

(5) $$index=50\cdot \frac{2^{level*2}-1}{3}+10\cdot 2^{level}\cdot IDY+IDX$$

The size and location of a sector are defined by (level, IDX, IDY). For example, the index of the sector (level:0, IDX:0, IDY:0) is 0. The latitude interval is −90°–−54°, and the longitude interval is −180°–−144°. The size of each interval is 36°. In the Unity3D editor, the *Sector* game object is created per sector under the *Globe* game object. It basically has a 3-D terrain model. The other layers of geospatial data in a sector are created as new game objects under the *Sector* game object. *The Sector Manager* class manages the *Sector* game objects. *GameObject:Find*() can access a game object in Unity3D. However, as the number of game objects increases, the object search time with the find function becomes slower. In *the Sector Manager* class, the *Dictionary* class variable is used to reference all *Sector* game objects and *Sector* class variables created in the platform. These approaches can minimize the time required to search *for Sector* game objects or *Sector* class variables.

### 3.2. Determination of the Rendering Sectors

**Figure 13:**
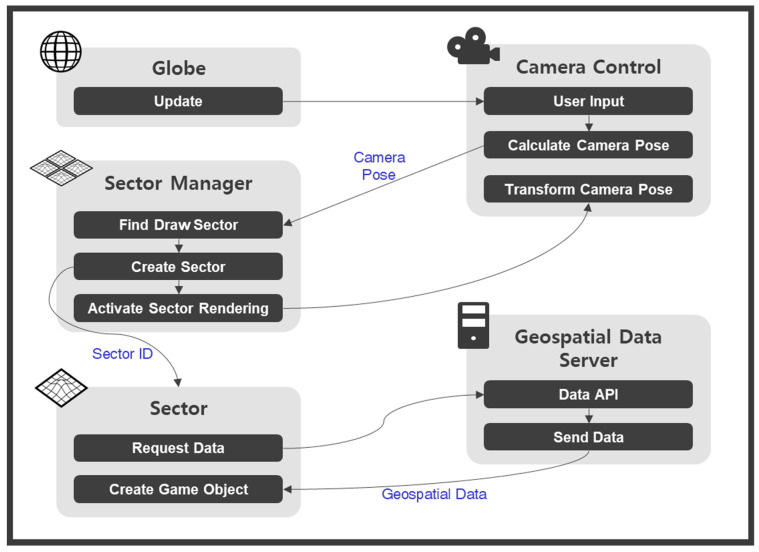
Task flow of the main loop.

Figure 13 shows the task flow of the main loop. The *Globe* class is the main class in the proposed platform. *The Camera Control* class calculates the position and orientation of the camera according to the user input. The *Sector Manager* class searches for the target sectors according to the calculated camera position and orientation. When the sector is determined as the rendering target, the *Sector* game object is created, if it does not exist. The *Sector* properties are defined as (level, IDX, IDY). The required data for sector creation are requested with (level, IDX, IDY) using the *Data API* from the *Geospatial Data Server*. Requesting data and creating game objects are performed asynchronously. Therefore, sector creation is delayed in relation to the frame that the sector is determined to be created. The rendering target sectors in this frame are selected according to whether a sector creation is completed. In advance of transforming the camera according to the user input, the rendering target sectors are determined from the calculated position and orientation of the camera. At the final step of the loop frame, the camera is transformed to the calculated position and orientation.

**Figure 14:**
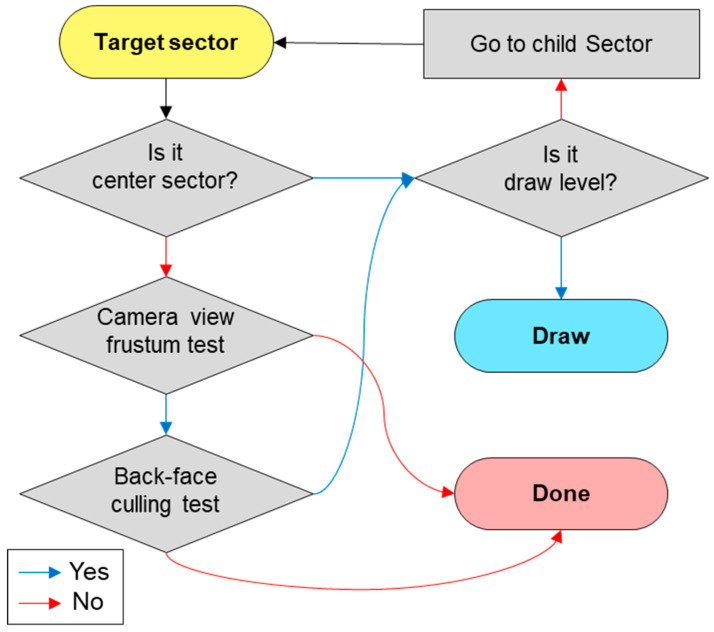
Task flow of the proposed three types of culling test.

In the platform implemented in Unity3D, among all of the 71.5 billion sectors, only approximately 60 or fewer sectors are rendered in a frame with a screen resolution of 1920 × 1080. The most time-consuming step in a loop frame is the search for sectors to be rendered. We propose a method for determining the rendering sectors based on the quadtree-based tile structure—specifically, whether the target sectors are rendered through three types of the culling test. Figure 14 shows the task flow of the culling test process.

The first step is to determine whether a sector is the central sector. This sector is, among the sectors through which the principal line of the camera passes, the closest to the camera. The shape of the sector located on the surface of the globe is a curved surface. When the size of the sector is too large, it may not pass the back-face culling test, which uses the four corner points and the center point vectors of a 3-D terrain model in a sector. Therefore, the closest sector through which the principal line of the camera passes is always rendered. The second step involves placing the target sector in the camera view frustum. The third step is the back-face culling test. The target sectors, except the center sector, must pass both the camera view frustum and back-face culling tests to enter the final verification process. Finally, if the level of the target sector is the same as the rendering level in (5), it is rendered in the current frame. If not, the child of the target sector becomes the target sector and the three culling tests are performed again. Instead of targeting a total of 71.5 billion sectors, the proposed method targets 50 sectors at level 0. It starts from all level 0 sectors and repeats the proposed test for the child sectors until it reaches the rendering level at every frame.

The process of rendering target sectors involves requesting data from the external geospatial data server. Two-dimensional or 3-D models are created as game objects using the downloaded data. At the initial stage of the loop frame, the enabled property of the mesh renderer components of all sectors is changed to false. When the sector is determined to be rendered, the enabled property is activated. The following rules are involved in the proposed rendering sector decision process.

- The parent sector is one level lower than the child sector. The parent sector contains the area of four child sectors.
- The child sectors are one level higher than the parent sector. The child sectors are divided by four from the parent sector.
- The created sector implies that data are requested and downloaded, and the sector is created as a game object in Unity3D.
- The child sectors can be created only when the parent sector is already created. Accordingly, all created sectors have the created parent sector except the level 0 sectors.
- When a new layer is added, all created sectors become a sector whose creation is not completed. The sectors are created by requesting data for the newly added layer from level 0.
- A sector can only be rendered if its neighboring child sectors, which have the same parent sector, are created, except for the level 0 sectors. If no child sector has been created, the parent sector is the rendering target.

**Figure 15:**
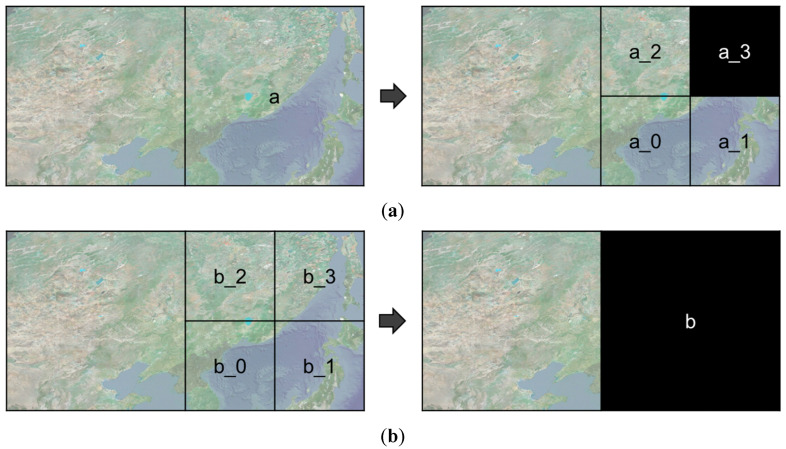
Examples of rendering failure: changes in rendering target sectors due to changes in position and orientation of the camera (**a**) the child sectors are rendered before the creation of all four child sectors; (**b**) the child sectors are created without the creation of a parent sector.

The rendering target sectors are changed according to the camera control. Depending on the network environment, sector creation is difficult to complete within 16.67 ms, which is a frame reference time of 60 fps. This implies that some sectors are not created and are displayed as black empty spaces, as shown in Figure 15. By following the above rules, it is possible to avoid unnatural visualizations. Figure 15a shows the child sector *a_3* of *a*, which is not created. Following the proposed rules, the child sector should be targeted only when all four child sectors that have the same parent sector have been created. Figure 15b shows a case in which parent sector *b* is not created. If all of the created child sectors have created parent sectors, *b* will be visualized normally.

**Figure 16:**
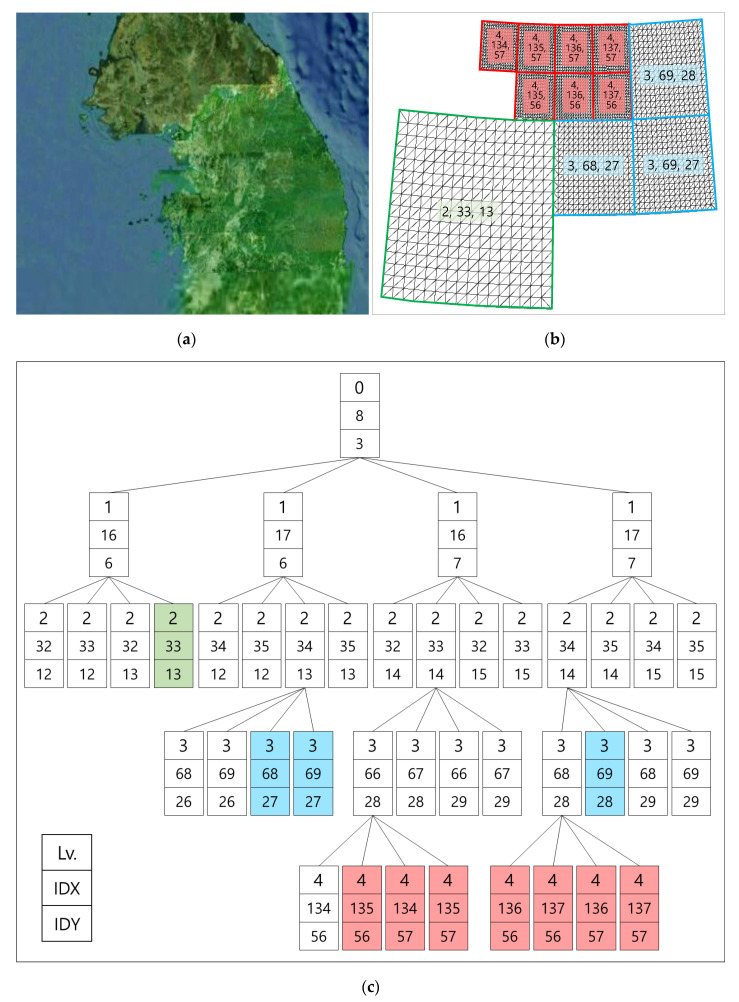
Rendering example of adjacent sectors and the quadtree structure: (**a**) rendering result for sectors in the game window; (**b**) wireframes of rendering sectors; (**c**) quadtree structure of rendering sectors (only colored sectors are rendered).

Figure 16 shows a rendering example of adjacent sectors of different levels. As it covers approximately 71.5 billion target sectors, only the rendering target sectors are activated according to the camera position and orientation. According to the decision rule of rendering sectors, all sectors except those at level 0 have a parent sector. A sector cannot be rendered before the same child sectors are created. Only the 11 colored sectors among the 41 sectors are rendered, as shown in Figure 16c.

## 4. Discussion

In a speed performance experiment, three types of computers were used: a desktop computer with an Intel^®^ 4.20-GHz Core™ i7 CPU, an NVIDIA^®^ GeForce^®^ GTX 1080Ti GPU, and wired network access; a notebook computer with an Intel^®^ 4.2-GHz Core™ i7-8650U CPU, an NVIDIA^®^ GeForce^®^ GTX 1060 GPU, wireless network access and a notebook computer with an Intel^®^ 4.2-GHz Core™ i7-8650U CPU, no GPU, an Intel^®^ HD Graphics 620 integrated graphics unit, and wireless network access.

Table 1 shows the performance results for each major step in the different types of environments. Taking into consideration the flexible network environment, the speed measurements were obtained at the frame at which the processes of downloading data using the DATA API from the external geospatial data server and generating downloaded data as a game object had been completed. The camera control step included the camera control by the user, calculation of the camera position and direction, calculation of the central longitude and latitude (coincident with the principal line of the camera and the terrain model), and other parameters related to the camera control. Our platform was able to perform at a rate of over 64.5 fps on the notebook computer without a GPU.

**Figure 17:**
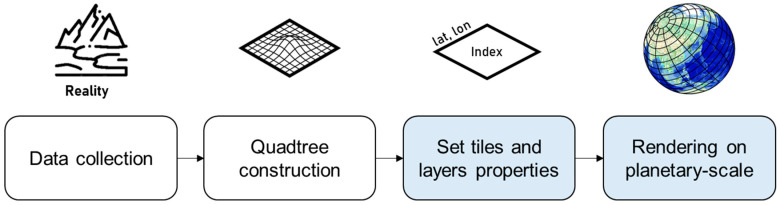
Development pipeline for digital twin city platform: the blue blocks show the steps followed in the proposed platform.

The proposed platform can be employed as a general digital twin city platform that can utilize geospatial data from various sources. Figure 17 shows the development pipeline employed to build a digital twin city platform using the proposed platform. First, digitized geospatial data are to be collected in the real city. Second, the digitized data must be generated in a tile unit with level-of-detail (LOD) on the basis of the quadtree structure [33]. All the 3-D data must then be transformed as per the proposed coordinate systems, as shown in Figure 8. In this study, we implemented a platform using VWorld data that involves a quadtree LOD built on the basis of the actual cities. The blue blocks in Figure 17 depict the steps followed in the proposed platform. The next step is to set the quadtree-based tile units and layer properties on the proposed platform. This involves setting the division units on the Earth’s surface and LOD steps, as shown in Figure 10 and Figure 11. In our study, we have defined a sector as a unit that groups various layers within a tile unit. When newly collected and quadtree-based, constructed, digitized city model data are set as a new layer within a sector, the proposed platform enables the geospatial data to be rendered on a planetary-scale.

Table 2 shows the differences between the platforms compared in the paper and the proposed platform. The purpose of our platform is to develop a general geospatial platform for supporting various type of geospatial data. Some cities are conducting 3-D geospatial data model constructions such as terrains, buildings, facilities, etc. They plan to establish a digital twin city in the future. There are limitations to developing a digital twin city platform based on web-based Google Earth or VWorld service sites. Additionally, even if a game engine-based game solution of Google Maps platform is used, the managing and rendering of large-scale tiled data with LODs in other formats is not supported.

## 5. Conclusions

In this study, we propose a planetary-scale geospatial open platform developed in a universal game engine environment. We describe how to organize and manage various types of geospatial data. The proposed method creates game objects in Unity3D based on quadtree tiles using VWorld geospatial data from an external data server. We propose a method for quickly searching the entire database, which spans over 30 TB and 71.5 billion tiles, to determine the rendering data. The experimental results provide a constant geospatial data visualization to the user and explain how to determine the rendering data. One can easily use the proposed 3-D geospatial platform by importing a small asset library into a Unity3D project. The proposed geospatial platform is available on the Asset Store, and has the potential to be widely used as a general geospatial platform for various purposes.

In future work, we will research a geospatial platform for the development of a digital twin that will display not only static data for city components, such as terrains, buildings, and facilities, but also movable dynamic data components, such as cars and pedestrians. This will require dynamic spatiotemporal data detection and storage management.

## Figures and Tables

**Table 1:** Results for each step of performance tests.

Step	Speed for Desktop with GPU (Graphics Processing Unit) (ms)	Speed for Notebook with GPU (ms)	Speed for Notebook without GPU (ms)
**Camera control**	0.0092	0.0123	0.0375
**Find draw sectors**	0.2202	0.2551	0.5930
**Activate draw sectors**	0.6741	0.8201	2.8769
**Frame rate (fps)**	6.0959 (164.04)	8.8838 (112.56)	15.4913 (64.55)

The resolution of the game window was 1024 × 768, and the screen resolution was 1920 × 1080. The speed results were measured with an average speed of 3,000 frames. The sizes of the graphic buffers were as follows: triangles, 111.8 k; vertices, 269.4 k; textures, 339.6 MB; 859 game objects; 430 sectors. The frame rate is the time of the main loop, including other steps.

**Table 2:** Comparison of geospatial platforms (accessed Oct. 19, 2020). The proposed platform does not provide its own geospatial data, but it was developed based on VWorld geospatial data. Our platform can substitute other geospatial data for VWorld data. LOD: level-of-detail.

	Google Earth [7]	VWorld Service Site [22]	Game Solution of Google Maps [15]	Proposed Platform
**Development environment**	web	web	Unity3D	Unity3D
**Scale**	planetary-scale	planetary-scale	city scale	planetary-scale
**Provide geospatial data**	yes	yes	yes	no (VWorld)
**Texture resolution of buildings**	low	high	low	-
**Supported locations**	global	South Korea, North Korea, and global (only terrains)	global	-
**Tiled data with LODs management**	yes	yes	no	yes
**External tiled** **data usage**	limit	limit	limit	support
**Price**	free	free	USD 200 (monthly usage)	free
